# Professional and Interprofessional Identity Formation in Healthcare Students During Placement in an Interprofessional Training Unit – A Multicentre Quantitative Study

**DOI:** 10.5334/pme.1649

**Published:** 2025-07-16

**Authors:** Saskia C. M. Oosterbaan-Lodder, Jan Jaap Reinders, Marco A. C. Versluis, Fedde Scheele, Rashmi A. Kusurkar

**Affiliations:** 1 Amsterdam Public Health, Quality of Care, Amsterdam, The Netherlands; 2 Amsterdam UMC location Vrije Universiteit, Research in Education, Amsterdam, The Netherlands; 3 Department of Research and Epidemiology, OLVG, Amsterdam, The Netherlands; 4 Research Group Healthy Ageing, Allied Health Care and Nursing, Hanze University of Applied Sciences, Groningen, The Netherlands; 5 Skills & Simulation Network Northern Netherlands, Groningen, The Netherlands; 6 Research Group Interprofessional Education (IPE), Lifelong Learning, Education & Assessment Research Network (LEARN), Research Institute SHARE, University of Groningen, University Medical Center Groningen, Groningen, The Netherlands; 7 Department of Obstetrics and Gynecology, University Medical Centre Groningen, Groningen, The Netherlands; 8 Athena Institute, faculty of science, Vrije Universiteit, Amsterdam, The Netherlands; 9 LEARN! Research Institute for Learning and Education, Faculty of Psychology and Education, VU University, Amsterdam, The Netherlands

## Abstract

**Introduction:**

Using Identity and Social Identity Theory as frameworks, we investigated the contribution of placement in an Interprofessional Training Unit (ITU) on a maternity ward to professional and interprofessional identity development, the interplay between these identities, and the association with some background factors.

**Methods:**

A prospective pre-post-intervention design was used. Before and after ITU placement, students in two Dutch hospitals participated in an online survey, comprising background characteristics, and scales measuring professional (PI) and interprofessional identity (IPI). PI and IPI scores between T0 and T1 were compared by means of a paired t-test. A two-way factorial ANOVA was used to test for differences between groups, based on background factors.

**Results:**

Out of 209 students, 179 students (89.9%) completed the pre- and post-placement surveys. We found no significant change in professional identity formation, but a significant increase in interprofessional identity formation across the placement for students from all three professions, with midwifery students having the highest increase. Students with higher PI scores had significantly higher IPI scores before placement compared to students with lower PI scores, without a significantly higher increase in IPI during the placement. No significant differences in IPI change were found for other variables.

**Discussion:**

We found that a one-week interprofessional placement significantly increased students’ interprofessional identity, with midwifery students showing the largest raise. Students with a higher PI also had a higher IPI before the ITU placement, suggesting a stronger PI is associated with a greater propensity for fostering IPI. Possible explanations for these findings are offered.

## Introduction

A growing body of research has highlighted the advantages of interprofessional education (IPE) to prepare future healthcare professionals (HCP) for Interprofessional Collaborative Practice [1,2,3]. One approach to implement IPE in a clinical healthcare setting involves students learning and working together in Interprofessional Training Units (ITUs) within hospital departments. Healthcare educators agree that healthcare students need opportunities to develop their professional identity (PI) [4,5,6] as well as learning to collaborate interprofessionally. Several authors have recently suggested that explicitly facilitating interprofessional identity (IPI), or extended professional identity, is recommended as a goal of these dedicated interprofessional placements [7,8], rather than just acquiring interprofessional competencies or attitudes. Developing an IPI is important since it is more central to the self and has the potential to improve interprofessional collaboration [9], due to identity-behaviour congruence—the idea that individuals are more likely to act in accordance with identities that are internalized and meaningful to them [10]. Furthermore, a strong PI and IPI can also increase professional wellbeing [11], ultimately contributing to reaching the quintuple aim of interprofessional education and collaboration [12,13]. The development of PI among health professions students is a complex, multifaceted process that involves acquiring knowledge, skills, values, and attitudes that align with their chosen profession. In the same way, development of an IPI could then be defined as a similar process but embedded in *interprofessional* healthcare. Cantaert et al. identified a significant gap in understanding how PI and IPI develop and influence each other, and proposed that combining perspectives from Social identity and Identity Theories could address this gap [14].

### Social Identity Theory and Identity Theory frameworks

Individuals simultaneously hold multiple social identities, including a PI and an IPI, whose salience depends on context and task relevance [15]. According to *Social Identity Theory*, individuals can form a sense of identity through their membership in a group, such as a particular professional community. This theory explains intergroup processes, distinguishing between ingroup and outgroup members [16]. When individuals move across different communities of practice within the landscape of practice, they must negotiate their identities. This negotiation involves reconciling the expectations, values, and norms of different communities, which may sometimes align but often conflict [17]. *Identity Theory* explains how individuals develop and manage their multiple social identities depending on the context, and how individuals manage possible ‘inner conflicts’ between their social identities [18]. In psychology, Social Identity Theory and Identity Theory represent two distinct yet complementary perspectives on identity [19]: the interpersonal perspective – which focuses on interaction between members of different groups – and the intrapersonal perspective. Currently, three theoretical frameworks have been developed to explain what interprofessional identity is and how it is formed. The Extended Professional Identity Theory (EPIT) [20] and Tong’s theoretical approach [21] conceptualize IPI as a three-dimensional construct, while Khalili’s dual identity [22] consists of four dimensions. Both EPIT and Tong’s framework align with Cameron’s social identity model, offering a strong foundation for measuring PI and IPI formation during ITU placements [23].

Previous research also shows that both identities can evolve and be activated by several variables. Students of mixed profession groups may be reluctant to compromise their professional priorities or adapt solutions that accommodate other professions, fearing it might diminish their professional distinctiveness [24]. Despite extensive research on the importance of students’ professional identity development [25,26], little is known about how healthcare students’ professional and interprofessional identities change following placement in an ITU [27], and if an overly strong professional identity can hamper IPI development [28]. Previous research by Tong et al. showed that nursing students maintained their IPI during a faculty-wide interprofessional first year program, while students from other professions did not. Their study also showed that IPI development is facilitated by means of high quality contact experiences with students from other professions, and by introductory IPE programs that provide students with opportunities to develop a clear understanding of their own professional roles [29]. Lindh Falk et al. found that medical students were significantly less positive than other healthcare students concerning the value of ITU, and that male students were significantly less positive than female students about how ITU placement contributed to their professional development [30]. This finding was attributed to male students identifying themselves more with their own professional group than female students, as a result of the male norm [31]. Wyatt et al. found that students who were considered underrepresented experienced a lack of immediate belongingness to the medical profession and struggled to integrate their ethnic and professional selves [32]. While some authors posit that PI development precedes IPI development [33], others suggest that a strong PI can be a barrier to interprofessional collaboration [34]. Although the interplay between professional identity and interprofessional identity is highlighted as an important research priority in recent literature [28,35], there remains a lack of studies that explore the potential dissonance between PI and IPI among students, particularly within ITUs [36].

As far as we know, the influence of a clinical ITU placement on PI and IPI of healthcare students has not been researched, nor how PI and IPI development during ITU placement are related to profession, gender, ethnic or cultural self-identification, and prior formal IPE experience. Understanding these relationships could provide educators with valuable insights in shaping the design and content of ITU placements, supporting more inclusive and impactful learning environments that support students from diverse professions in developing their PI and IPI.

Therefore, the research questions of our study were:

1. What is the effect of a one-week placement in a clinical ITU on students’ professional identity (PI) and interprofessional identity (IPI);
2. Is there a different effect of the ITU placement on students’ identities between students from different professions, gender, ethnic or cultural identity, site, and high or low professional identification before placement.

We had the following hypotheses:

**H1a**. Professional identification does not change significantly across an ITU placement.

**H1b**. Interprofessional identification for students increases across an ITU placement.

**H2**. The influence of ITU placement on PI and IPI of students differs for background variables.

## Methods

### Research design

This study used a multicentre prospective pre-post intervention design, conducted in two teaching hospitals in the Netherlands, with site 1 located in a secondary care hospital, and site 2 in a tertiary care hospital.

### Research setting and sampling

During a one-week mandatory placement in an ITU, medical, midwifery and nursing students were learning from, with, and about each other’s roles and responsibilities within an ITU on the maternity ward of two Dutch teaching hospitals. The ITU in Amsterdam (site 1) was instituted in October 2016, and the ITU in Groningen (site 2) in October 2022. The ITUs covered a part of the single hospital rooms on the maternity ward. The students were responsible for planning, delivering, and evaluating the treatment of mothers and their new-borns within these ITUs during the daytime shift. They were supervised by trained nursing and midwifery tutors [37]. One week prior to their ITU placement, students received study participant information, after which they were invited through email to complete an online survey before ITU placement (T0) and after ITU placement (T1). Students could complete the survey within one week after their placement. On site 1, students were guided by midwifery and nursing tutors during their activities, while on site 2, medical and midwifery students were guided mainly by midwives, whereas the nursing students were guided mainly by nurses. Furthermore, nursing students on site 2 participated in the placement one to two days per week for two months, whereas nursing students on site 1 participated for one week. Surveys were distributed between September 2023 and July 2024 to all students that participated in the ITU. Completing the survey was considered to be the students’ consent to participate.

### Data collection and measures

The quantitative data of students’ PI and IPI were obtained by means of an online survey distributed before and after ITU placement. The survey at T0 included collection of the following demographic data: age, profession, gender, self-identified ethnic or cultural identity, prior formal IPE experience, and site. The surveys at T0 and T1 included two scales, measuring Professional Identity and Interprofessional Identity. Both scales were used after obtaining permission from the original researchers, and translated into Dutch following established guidelines for cross cultural adaptation [38], after which they were tested – before September 2023 – with a sample of the target population. Minor adjustments were made to ensure clarity. Data were collected through CastorEDC [39], a secure cloud-based electronic data capture (EDC) platform designed for clinical research, in compliance with Good Clinical Practice.

In this study, professional identity (PI) was measured using Tong’s adapted Three-Factor Model of Social Identity Scale (TFSIS), based on the Three-Factor Model of Social identity scale [23]. This scale was used because this allowed us to measure PI of students from different professions with the same scale. In this adjusted version, references to ‘ingroup member(s)’ had been replaced with references to ‘members of my profession’ [29]. This instrument consists of three subscales with four items per subscale, which were scored on a 6-point Likert scale (from strongly disagree to strongly agree). Examples of items are as follows: “I have a lot in common with other people from my profession” (ingroup ties), “Generally, I feel good when I think about myself as being part of this profession” (ingroup affect). “In general, being part of this profession is an important part of my self-image” (centrality). In a previous study using this instrument, internal consistency of the measurement with the overall scale was 0.81 [8,29].

Since several studies have supported many propositions of EPIT [40,41,42,43,44], in this study IPI was measured using the Extended Professional Identity Scale (EPIS), based on EPIT. This measurement instrument consists of 12 items, three subscales and four items per subscale, which were scored on a 5-point Likert scale (from strongly disagree to strongly agree). In a previous study, internal consistency of the measurement with the overall scale was 0.89, and 0.79, 0.81, and 0.80 of the subscales, respectively [44]. Examples of items are as follows: “I like learning about other health professions” (interprofessional belonging), “I prefer working with others in an interprofessional team” (interprofessional commitment), and “All members of an interprofessional team should be involved in goal setting for each patient” (interprofessional beliefs) (supplement A). The dimensions of the TFSIS – ingroup ties, ingroup affect, and centrality – align conceptually with the dimensions of EPIS – interprofessional belonging, interprofessional commitment and interprofessional beliefs [23].

### Data analysis

A total of 209 cases from T0 were merged from CastorEDC into one IBM SPSS (v. 29.0) data file. Of these 209, 198 were available for analysis. Cases were excluded if measures for PI or IPI were missing (N = 10). After matching participants by email address, data from T0 and T1 were merged into one data file. Then email addresses were replaced with identification numbers. The Likert ordinal variables, with six and five categories respectively, were used as continuous variables [45]. Descriptive statistics were reported as frequencies (relative frequency: %) for categorical data, mean and standard deviation (SD) for normally distributed data. Differences in PI and IPI scores at T0 were tested with one-way ANOVA for different professions, and unpaired t-tests for other variables. Differences in mean total scores of the TFSIS and EPIS between T0 and T1 were tested by means of a paired t-test, after testing for normality of the data, using the Kolmogorov-Smirnov test. A two-way factorial ANOVA was used to examine differences between groups, based on profession, gender, self-identified ethnic or cultural identity, prior formal IPE experience, site, and high or low PI before placement; post-hoc comparisons were conducted using the Bonferroni correction. Partial eta squared (η^2^) was computed as a measure of effect size for ANOVA analyses.

### Ethical considerations

Ethical approval for the study was obtained from the Netherlands Association for Medical Education (file 2023.4.5) and from the Scientific research Committee in OLVG (ACWO research no WO 23.086). Students were approached by mail with information about the aim and purpose of the study. The information also stated that participation was completely voluntary, with no incentives, and that participants could withdraw from the study at any time without giving any reason for their withdrawal, up to two months after completing the survey. Researchers had no hierarchal relationship with the students.

## Results

### Sample characteristics

A total of 209 students participated in the ITU and were invited to participate in the survey. Of these, 198 (94.7%) completed the survey at T0 and 179 (89.9%) completed the survey at T1 as well. There was no missing data in these 179 surveys. Table 1 displays the participants’ demographics.

**Table 1:** Distribution of participants by age, profession, gender, self-identified ethnic or cultural identity, prior formal IPE experience, and site at T0 (N = 198) and T1 (N = 179).


		NUMBER OF PARTICIPANTS	

		T0 (N = 198)	T1 (N = 179)

Age (years)	<25 ≥25	157 (79.3%) 41 (20.7%)	141 (78.7%) 38 (21.3%)

Profession	Medical Midwifery Nursing	107 (54.0%) 70 (35.4%) 21 (10.6%)	98 (54.7%) 63 (35.2%) 18 (10.1%)

Gender	Female Male	161 (81.3%) 37 (18.7%)	146 (81.6%) 33 (18.4%)

Self-identified ethnic or cultural identification	Dutch Non-Dutch	169 (85.4%) 29 (14.6%)	152 (84.9%) 27 (15.1%)

Prior formal IPE experience*	No Yes	140 (70.7%) 58 (29.3%)	123 (68.7%) 56 (31.1%)

Site	site 1 site 2	113 (57.1%) 85 (42.9%)	109 (60.9%) 70 (39.1%)


*Defined as: students from different healthcare professions were learning with, from and about each other, during bachelor or a prior interprofessional placement.

On average, professional identity, measured by TFSIS score, of those who completed the survey at T0 only (N = 19; M = 4.56, SD = 0.59) was not significantly different (p = 0.452) from that of those who completed the survey at T0 and T1 (N = 179; M = 4.58, SD = 0.54), Cohen’s d = 0.55, 95% CI [–0.50, 0.44]. Interprofessional identity, measured by EPIS score, of those who completed the survey at T0 only (N = 19; M = 4.04, SD = 0.47) was also not significantly different (p = 0.321) from that of those who completed the survey at T0 and T1 (N = 179; M = 4.00, SD = 0.42), Cohen’s d = 0.43, 95% CI [–0.36, 0.59]. (Cohen, 2013). It is reasonable to consider that the results of students that completed the survey at T0 and T1 are representative of all students on the ITU, but caution is warranted because of the small T0-only sample.

All further analyses were conducted for students that completed surveys at T0 and T1.

### 1. Changes in professional and interprofessional identity strengths across ITU placement

Internal consistency for the measurement with the PI scale (TFSIS) at T0 was α = 0.71, and at T1 α = 0.76. Internal consistency for the measurement with the IPI scale (EPIS) at T0 was α = 0.82, and at T1 was α = 0.88, implying that the measurement of PI and IPI was reliable (Evers, Lucassen, Meijer, & Sijtsma, 2015; Nunnally, 1994). After testing for normality, a paired-samples t-test was conducted to compare PI and IPI scores at T0 and T1. There was no significant difference in the PI scores between T0 (M = 4.59) and T1 (M = 4.64), t(178) = 1.572, p = 0.118, 95% CI [–0.01473, 0.13019]. However, there was a significant increase in the IPI scores from T0 (M = 4.00) to T1 (M = 4.10), t(178) = 3.498, p < 0.001, 95% CI [0.04586, 0.16457] (Table 2).

**Table 2:** Mean scores and standard deviations on professional and interprofessional identity measures at the start (T0) and at the end (T1) of the ITU placement (N = 179).


	N	T0 MEAN (SD)	T1 MEAN (SD)	MEAN DIFFERENCE T1–T0 (95% CI FOR DIFF)	(TWO-SIDED) p-VALUE

Professional Identity (TFSIS)					

Total	179	4.59 (0.54)	4.64 (0.58)	0.06 (–0.015;0.130)	0.118

Subscale ingroup ties		4.84 (0.81)	4.97 (0.70)	0.13 (0.025;0.238)	0.016

Subscale ingroup affect		5.22 (0.67)	5.18 ((0.68)	–0.05 (–0.143;0.048)	0.329

Subscale centrality		3.67 (0.93)	3.76 (0.97)	0.09 (–0.028;1.501)	0.135

Interprofessional Identity (EPIS)					

Total	179	4.00 (0.42)	4.10 (0.48)	0.11 (0.046; 0.165)	<0.001

Interprofessional belonging		4.26 (0.54)	4.36 (0.55)	0.10 (.018; 0.189)	0.018

Interprofessional commitment		3.72 (0.60)	3.87 (0.61)	0.16 (0.040;0.075)	<0.001

Interprofessional beliefs		4.01 (0.52)	4.07 (0.60)	0.06 (0.040; 0.136)	0.152


The PI Ingroup Ties subscale score increased significantly from T0 to T1 (M = 0.13, t(178) = 2.43, p = 0.016), while the PI Centrality (M = 0.09, p = 0.135) and PI Ingroup Affect subscale scores (M = –0.05, p = 0.329) did not differ significantly between T0 and T1. There was significant increase in the IPI interprofessional commitment (M = 0.16, t(178) = 3.83, p < 0.001) and IPI Belonging subscale scores (M = 0.10, p = 0.018) from T0 to T1. No significant difference was found for IPI Beliefs subscale score between T0 and T1 (M = 0.06, p = 0.152).

### 2. Changes in professional and interprofessional identity strengths across ITU placement between students

***Before placement***, significantly higher PI scores were found for nursing students (F(2,176) = 3.98, p = 0.020), female students (t(177) = 1.859, p = 0.032, two-tailed, MD = 0.19382 (95% CI: –0.01195 to 0.39959, Cohen’s d = 0.54095 (95% CI: 0.358 to –0.022), and students identifying as Dutch (t(177) = 2.098, p = 0.037), MD = 0.23640 (95% CI: 0.01403 to 0.45876), Cohen’s d = 0.540 (95% CI: 0.026 to –0.849). Furthermore, before placement, significantly lower IPI scores were found for midwifery students (F(2,176) = 4.15, p = 0.017). Although there was no significant difference in total IPI scores before placement (p = 0.051), female students scored significantly higher than male students on the IPI subscale belonging ((t(177) = 2.127, p = 0.035) with a mean difference of 0.218 (95% CI 0.016 to 0.420). And although there were no significant differences in IPI scores (t(177) = –1.085, p = 0.279, two-tailed), students identifying as non-Dutch had significantly higher interprofessional commitment scores (t(177) = –2.247, p = 0.026, two-tailed). Results from the analyses are shown in Table 3.

**Table 3:** Mean PI and IPI scores before (T0) and after (T1) ITU placement, with item-scores on the Likert scale from 1 to 6 for PI, and item scores on the Likert scale from 1 to 5 for IPI.


		N	T0 MEAN (SD)	T1 MEAN (SD)	MEAN DIFFERENCE T1–T0 (95% CI FOR DIFFERENCE)	(TWO-SIDED) p-VALUE FOR TIME (F-STATISTIC)	(TWO-SIDED) p-VALUE FOR INTERACTION BETWEEN GROUP AND IDENTITY (F-STATISTIC)

Professional Identity (TFSIS)							

Total		179	4.59 (0.54)	4.64 (0.58)	0.06 (–0.015; 0.130)		

Profession	Medical Midwifery Nursing	98 63 18	4.52 (0.53) 4.58 (0.57) 4.90 (0.43)	4.59 (0.56) 4.60 (0.61) 5.01 (0.45)	0.07 (–0.024; 0.174) 0.02 (–0.110; 0.144) 0.11 (–0.119; 0.332)	0.854 (0.034)	0.698 (0.360)

Gender	Female Male	146 33	4.61 (0.55) 4.42 (0.48)	4.65 (0.60) 4.58 (0.48)	0.03 (–0.049;0.117) 0.16 (0.018;0.305)	0.040 (4.298)	0.179 (1.181)

Ethnic or cultural identification	Dutch Non-Dutch	152 27	4.61 (0.55) 4.38 (0.50)	4.64 (0.55) 4.61 (0.72)	0.03 (–0.049;0.103) 0.23 (0.010;0.453)	0.012 (6.449)	0.046 (4.048)

Prior formal IPE experience	No Yes	123 56	4.58 (0.53) 4.57 (0.57)	4.65 (0.58) 4.60 (0.58)	0.08 (–0.020;0.170) 0.02 (–0.087; 0.126)	0.235 (1.421)	0.482 (0.496)

Site	OLVG UMCG	109 70	4.58 (0.51) 4.57 (0.60)	4.64 (0.56) 4.64 (0.58)	0.05 (–0.038; 0.143) 0.07 (–0.058; 0.189)	0.119 (2.455)	0.866 (0.028)

PI before placement	Low PI High PI	90 89	4.15 (0.36) 5.01 (0.29)	4.37 (0.56) 4.91 (0.45)	0.22 (0.117; 0.324) –0.11 (–0.198; –0.016)	0.103 (2.679)	<0.001 (22.2023)

Interprofessional Identity (EPIS)							

Total		179	4.00 (0.42)	4.10 (0.48)	0.11 (0.046; 0.165)		

Profession	Medical Midwifery Nursing	98 63 18	4.05 (0.45) 3.88 (0.34) 4.13 (0.42)	4.10 (0.56) 4.09 (0.39) 4.18 (0.33)	0.05 (–0.043; 0.138) 0.21 (0.129; 0.291) 0.05 (–0.097; 0.199)	0.007 (7.412)	0.035* (3.406)

Gender	Female Male	146 33	4.03 (0.42) 3.87 (0.43)	4.14 (0.47) 3.91 (0.49)	0.12 (0.051; 0.185) 0.05 (–0.085; 0.181)	0.034 (4.583)	0.367 (0.818)

Ethnic or cultural identification	Dutch Non-Dutch	152 27	3.98 (0.43) 4.08 (0.41)	4.09 (0.48) 4.19 (0.47)	0.10 (0.039;0.170) 0.11 (–0.036; 0.252)	0.012 (6.372)	0.969 (0.002)

Prior formal IPE experience	No Yes	123 56	4.03 (0.40) 3.92 (0.47)	4.16 (0.44) 3.97 (0.55)	0.13 (0.066;0.195) 0.05 (–0.078; 0.179)	0.006 (7.779)	0.221 (1.506)

Site	OLVG UMCG	109 70	4.02 (0.45) 3.96 (0.38)	4.12 (0.51) 4.06 (0.48)	0.10 (0.024;0.184) 0.11 (0.018; 0.196)	<0.001 (11.667)	0.959 (0,003)

PI before placement	Low PI High PI	90 89	3.92 (0,42) 4.08 (0.41)	4.05 (0.52) 4.15 (0.44)	0.13 (0.032; 0.233) 0.08 (0.013; 0.141)	<0.001 (12.189)	0.365 (0.826)


*No difference between professions on the post-hoc scores.

The *effect of ITU placement* on PI across all *professions* was not statistically significant, F(1,176) = 1.986, p = 0.161, whereas the effect on IPI was statistically significant (F(1,176) = 7.41, p = 0.007, partial η^2^ = 0.040). Although a significant difference in IPI increase between professions was found (F (2,176) = 3.41, p = 0.035, partial η^2^ = 0.037), post-hoc comparisons using Bonferroni tests did not reveal statistically significant differences among the professions, maybe due to the low sample size of nursing students. Midwifery students had the greatest increase in IPI across the ITU placement, with MD = 0.21 (95% CI: 0.129; 0.291).

There was no significant difference in PI score or subscale scores between *male and female students* across the placement (F(2,176) = 1.818, p = 0.179). There was a significant difference in effect of the ITU placement on total IPI scores (F (1,176) = 4.583, p = 0.034, partial η^2^ = 0.025), without a significant difference between male and female students (F(2,176) = 0.818, p = 0.367, partial η^2^ = 0.005). However, female students did have a significantly greater increase than male students in interprofessional beliefs score (F(1,176) = 4.243, p = 0.041, partial η^2^ = 0.023). The observed power was 0.535, suggesting that results were not due to random chance.

Across placement, *students identifying as non-Dutch* had a significantly greater increase in PI (F(1,177) = 4.05, p = 0.046, partial η^2^ = 0.022), with a significantly higher increase in ingroup ties than students identifying as Dutch (F(1,177) = 7.75, p = 0.006, partial η^2^ = 0.042, observed power = 0.791).

There was no significant difference in increase in IPI between students identifying as Dutch or non-Dutch (F(1,177 = 0.002, p = 0.969). There was a significant main effect of the placement on interprofessional belonging (F(1,177) = 7.046, p = 0.009, partial η^2^ = 0.038), and on interprofessional commitment (F(1,177) = 4.633, p = 0.033, partial η^2^ = 0.026), without a significant difference between the two groups.

Across placement, IPI increase was significant (p = 0.006, partial η^2^ = 0.042, observed power of 0.792). On subscales, a significant main effect of the placement was found only on interprofessional commitment, F(1,177) = 10.748, p 0.001, partial η^2^ = 0.057), without significant effects on the other subscales. There was no significant difference in IPI increase between *students with or without prior formal IPE experience* (F(1,177) = 1.51, p = 0.221).

A significant effect of the placement was found on ingroup ties (F(1,177) = 4.888, p = 0.028, partial η^2^ = 0.027), without a significant difference between both sites (F(1,177) = 0.530, p = 0.467, partial η^2^ = 0.003). There was no significant difference of the ITU placement on IPI between the *two sites* (p = 0.959, with a partial η^2^ = 0.050).

Students were divided into two groups, with lowest (N = 90) and highest (N = 89) PI at T0, where we found a significantly *higher IPI score for students with a high PI* score (t(177) = 2.56, p = 0.011, MD = 0.159 (95% CI: 0.282, 0.036). There was a significant effect of the placement on IPI, F(1, 177) = 12.19, p < 0.001, partial η² = 0.064, indicating a small to medium effect size. However, there was no significant difference in IPI across ITU placement between students with a low PI (N = 90) and high PI (N = 89), F(1, 177) = 0.83, p = 0.365, partial η² = 0.005. Observed power was high for the main effect (0.935) but low for the interaction effect (0.148).

## Discussion

To our knowledge this is the first study to quantitatively measure changes in professional and interprofessional identities in medical, midwifery and nursing students during a one-week placement in an interprofessional training unit (ITU) on a maternity ward in a secondary and tertiary hospital. It also measured the interplay between professional and interprofessional identities and examined how these identities related to other social identities, including gender, self-identified ethnic or cultural identity, prior formal interprofessional education (IPE) experience.

*Professional identity* did not change significantly across the ITU placement for the overall group of students, confirming our hypothesis. However, professional ingroup ties showed a significant increase. This may reflect students expanding their social networks or group memberships during the placement. When ITU placements clarify students’ roles and their contributions to the team, they are more likely to develop a stronger social identity within their professional group. Such clarity enhances their sense of ingroup ties, or belonging to their professional group [16].

*Interprofessional identity* increased significantly across the ITU placement. The significant increase in interprofessional belonging may be explained by positive feedback and support from peers and tutors during their ITU placement. Since the dimension of professional ingroup ties aligns conceptually with interprofessional belonging [23], our findings support the fact that interprofessional placements can support both professional and interprofessional belongingness [46]. The significant increase in interprofessional commitment may have been fostered by increased insight into roles and responsibilities of other professions and recognition from peers from other professions. The frequent interprofessional contact between students and tutors on the ITU could have fostered this sense of belonging and commitment to an interprofessional group, leading to favorable attitudes. This is in line with recent research reporting increased interprofessional identification across an IPE teaching module among students in health professions education [47]. It is noteworthy that this effect was seen after only a one-week mandatory interprofessional placement.

Midwifery students had the lowest interprofessional identity at the start of the ITU placement, maybe due to less social interaction between various professions in their primary maternity care in the years prior the ITU placement. Medical and nursing students interact with other professions during clinical rotations before their ITU placement, fostering construction of a sense of the ‘other’, informing their developing sense of themselves as HCPs [24]. Nursing students, through prior informal interprofessional interactions in maternity care before the ITU placement, had opportunities to compare professional realities with theoretical learning [48], reinforcing their understanding of client-centred care and likely contributing to their higher professional and interprofessional identity compared to medical and midwifery students. Midwifery students showed the largest increase in interprofessional identity, possibly because the ITU placement allowed them to navigate interprofessional tension by showcasing midwifery training and engaging in interprofessional learning [49].

Our finding that before ITU placement female students had significantly higher scores on the subscale interprofessional belonging might reflect the fact that female students expected to feel valuable and included within maternal care, in line with research on male students demonstrating they are less keen on undergoing maternity training [50]. The significantly greater increase in interprofessional beliefs across the female students’ placement compared to male students is in line with previous research showing that female students have a more positive view of teamwork [51] and higher perceived relevance of teamwork [52]. Belonging to a specific profession within an interprofessional community likely depends on professional beliefs about the profession’s role within a broader network of various professions. This position might be either profession-centred or more holistic, with females more often favouring a holistic approach, in line with research by Reinders et al. [15] and Atwa et al [53]. Male students may have perceived the female-centred maternity ward as less supportive of their identity than their female peers. Variations in how identity attributes are valued across contexts could lead to different outcomes.

Students identifying as non-Dutch showed significantly lower professional identity before the placement but a greater increase across the placement, particularly in ingroup ties. No difference was found in interprofessional identity increase between the two groups. This could be due to minority students’ finding that expectations from the majority perspective misalign with their values and orientations [54]. Dutch students, possibly aligning more with individualistic and autonomous worldviews, may more easily develop a positively distinct social identity compared to non-Dutch students, who may lean towards collectivistic perspectives, in line with previous research [55].

Students with prior formal IPE experience showed lower interprofessional identity before the placement, possibly because they might not have been developmentally ready to grasp the relevance of interprofessional education in the early stages of their education [29], or because they may have encountered a hidden curriculum, with negative role models or institutional practices [56]. Encouragingly, interprofessional identity increased after a one-week placement for all students, emphasizing the value of positive interprofessional role models. We found no differences in interprofessional identity increase between the two sites, suggesting that both contexts effectively fostered interprofessional identity, consistent with Heise et al. [18]. However, outcomes might differ on wards with patients with different health challenges.

Students with a higher professional identity also had a higher interprofessional identity before the ITU placement, suggesting that a stronger professional identity is associated with a greater propensity for fostering IPI, in line with the Extended Professional Identity Theory (EPIT) [15]. However, we found no difference in interprofessional identity increase across the ITU placement between students with high or low pre-placement professional identity, suggesting that a high professional identity posits no barrier to develop an interprofessional identity, when educators successfully bridge professional differences [34]. Personalized, student-centred interprofessional learning, enriched with reflection and mentoring can nurture professional identity formation and activation, as was also mentioned by Wald et al. [26], whereas supporting awareness about and reflection on diversity and inclusiveness between students, and between students and tutors, could foster interprofessional identity [57].

## Limitations and future research

The ITU placement was mandatory for the students, which minimizes the possibility of IPI scores having been influenced by personal interest or motivation to learn and collaborate interprofessionally. The survey completion rate of nearly 90% ensured a sufficiently large sample at both time points, providing adequate statistical power for the analysis of within-subject differences and interactions between groups. A limitation of our study was the small sample size of nursing students, which might have influenced the results.

In an ITU on a different ward, the context, patients, and tutors could vary, potentially leading to different outcomes. Measuring students’ interprofessional identity development across their undergraduate and graduate training could provide valuable insights for educators and administrators regarding the effects of interprofessional education (IPE) interventions within healthcare curricula. Furthermore, research on contextual triggers that activate interprofessional identification could inform faculty development of tutors guiding students on an ITU as well as in guiding students during their whole undergraduate (inter)professional development.

## Conclusions

In this study we found that a one-week interprofessional placement significantly increased students’ interprofessional identity, with midwifery students showing the largest rise in interprofessional identity. Students with higher PI did not exhibit a significantly larger increase in interprofessional identification across the placement but showed a higher interprofessional identity before the ITU placement, suggesting that a stronger PI is associated with a greater propensity for fostering IPI.

## Data Accessibility Statement

The dataset from this study is available from the corresponding author on request.

## Additional File

The additional file for this article can be found as follows:

10.5334/pme.1649.s1 Supplement A. Introduction questions.

## Author Information

Saskia C M Oosterbaan, MD, PhD student on Interprofessional Education (IPE), is a certified senior medical educator and IPE program coordinator at Teaching Hospital OLVG, Amsterdam, the Netherlands. She has a background in pediatrics and youth healthcare.

Jan Jaap Reinders, PhD, is an organisational psychologist specialized in interprofessional collaboration, education and integrated care.

Marco AC Versluis, MD, PhD, is a practicing gynaecologist and obstetrician as well as IPE coordinator and researcher in UMCG, Groningen, the Netherlands.

Fedde Scheele, PhD, MD, is a Professor of Health Systems Innovation and Education at the Amsterdam UMC and at the Athena Institute, faculty of science, of the Vrije Universiteit Amsterdam, the Netherlands. He is a non-practicing gynaecologist, a certified trainer for pre- and postgraduate clinical education and the dean of the Academic Centre for Dentistry Amsterdam (ACTA).

Rashmi A Kusurkar, PhD, MD, FAMEE, is a Professor on Inclusion and Motivation in Health Professions Education and Research Programme Leader at Amsterdam UMC, location Vrije Universiteit, Research in Education, Amsterdam, the Netherlands.
