# Supplementary material for: Professional and Interprofessional Identity Formation in Healthcare Students During Placement in an Interprofessional Training Unit – A Multicentre Quantitative Study
Source: Perspect Med Educ. 2025 Jul 16;14(1):399–410. doi: 10.5334/pme.1649 (PMC12273683; doi:10.5334/pme.1649)
Supplement: Supplement A. — Introduction questions. [file pme-14-1-1649-s1.pdf]

Supplement A

Introduction questions, please tick and fill in what applies

1. Age: 20-30 years ☐, 30-40 years ☐ , 40-50 years ☐, 50-years ☐

2. What is your profession?
   medical student OBGYN ☐, medical student paediatrics ☐, 2nd year midwifery student ☐, 3rd year midwifery student ☐ nursing student ☐ other

3. Gender: male ☐ female ☐ other ☐

4. With which ethnic or cultural groups do you identify yourself?
   ☐ Dutch
   ☐ non-Dutch, namely……

5. In which hospital is your ITU-placement located?
   OLVG ☐
   UMCG ☐

7. Did you have previous formal IPE experience?
   No ☐
   Yes ☐, namely. ……………………………………………………………………………………………

Adapted Three-Factor Model of Social Identity Scale (TFSIS)

1 = strongly disagree
2 = moderately disagree
3 = slightly disagree
4 = slightly agree
5 = moderately agree
6 = strongly agree

**<u>Ingroup ties (perceptions of similarity and belonging to group members)</u>**						
I have a lot in common with other people from my profession.	1☐	2☐	3☐	4☐	5☐	6☐
I feel strong ties with other people from my profession.	1☐	2☐	3☐	4☐	5☐	6☐
I find it difficult to form a bond with other people from my profession.	1☐	2☐	3☐	4☐	5☐	6☐
I don’t feel a sense of being “connected” with other people from my profession.	1☐	2☐	3☐	4☐	5☐	6☐
**<u>Centrality (amount of time spent thinking about being a group member)</u>**						
I often think about the fact that I am part of this profession.	1☐	2☐	3☐	4☐	5☐	6☐
Overall, being part of this profession has very little to do with how I feel about myself.	1☐	2☐	3☐	4☐	5☐	6☐
In general, being part of this profession is an important part of my self-image.	1☐	2☐	3☐	4☐	5☐	6☐
The fact that I am a part of this profession rarely enters my mind.	1☐	2☐	3☐	4☐	5☐	6☐
**<u>Ingroup affect (positivity of feeling associated with members of the group)</u>**						
In general, I’m glad to be part of this profession.	1☐	2☐	3☐	4☐	5☐	6☐
I often regret that I am part of this profession.	1☐	2☐	3☐	4☐	5☐	6☐

I don’t feel good about being part of this profession.	1☐	2☐	3☐	4☐	5☐	6☐
Generally, I feel good when I think about myself as being part of. this profession	1☐	2☐	3☐	4☐	5☐	6☐

Note. With permission, we used the adjusted version by Tong et al. (https://doi.org/10.1016/j.nedt.2019.104328) of the adapted version of The professional identity measure (which was adapted with permission from Cameron, J. E. (2004). A three-factor model of social identity. Self and identity, 3(3), 239-262. doi:10.1080/13576500444000047

Extended Interprofessional Identity Scale (EPIS)

1 = strongly disagree
2 = disagree
3 = neutral
4 = agree
5 = strongly agree

**<u>Interprofessional belonging</u>**					
I like meeting and getting to know people from other health professions.	1☐	2☐	3☐	4☐	5☐
I feel a strong attachment toward interprofessional teams comprising cross-disciplinary health professionals.	1☐	2☐	3☐	4☐	5☐
I like learning about other health professions.	1☐	2☐	3☐	4☐	5☐
I enjoy learning and collaborating with people from other health professions.	1☐	2☐	3☐	4☐	5☐
**<u>Interprofessional commitment</u>**					
I would be very happy to spend the rest of my career with an interprofessional team.	1☐	2☐	3☐	4☐	5☐
I prefer working with others in an interprofessional team.	1☐	2☐	3☐	4☐	5☐
I identify myself with other members of an interprofessional team.	1☐	2☐	3☐	4☐	5☐
I am proud to be a part of an interprofessional team.	1☐	2☐	3☐	4☐	5☐
**<u>Interprofessional beliefs</u>**					
Joint clinical decision-making should be an important part of interprofessional collaboration.	1☐	2☐	3☐	4☐	5☐
All members of an interprofessional team should be involved in goal setting for each patient.	1☐	2☐	3☐	4☐	5☐
Interprofessional team members should jointly agree to communicate plans for patient care.	1☐	2☐	3☐	4☐	5☐
When care decisions are made, the interprofessional team members should strive for consensus on planned processes	1☐	2☐	3☐	4☐	5☐

Reinders, J.J., Lycklama À Nijeholt, M., Van Der Schans, C.P. & Krijnen, W.P. (2020) The development and psychometric evaluation of an interprofessional identity measure: Extended Professional Identity Scale (EPIS), Journal of Interprofessional Care.
